# Lamins in Lung Cancer: Biomarkers and Key Factors for Disease Progression through miR-9 Regulation?

**DOI:** 10.3390/cells7070078

**Published:** 2018-07-16

**Authors:** Julien Guinde, Diane Frankel, Sophie Perrin, Valérie Delecourt, Nicolas Lévy, Fabrice Barlesi, Philippe Astoul, Patrice Roll, Elise Kaspi

**Affiliations:** 1 Aix Marseille Université, INSERM, MMG, 13385 Marseille, France; julien.guinde@ap-hm.fr (J.G.); sophie.perrin@univ-amu.fr (S.P.); valeriedelecourt22@gmail.com (V.D.); 2 APHM, Hôpital Nord, Department of Thoracic Oncology—Pleural Diseases—Interventional Pulmonology, CEDEX 5, 13385 Marseille, France; pastoul@ap-hm.fr; 3 Aix Marseille Université, APHM, INSERM, MMG, Hôpital la Timone, Service de Biologie Cellulaire, 13385 Marseille, France; diane.frankel@univ-amu.fr (D.F.); patrice.roll@univ-amu.fr (P.R.); 4 ProGeLife, 13385 Marseille, France; 5 Aix Marseille Université, APHM, INSERM, MMG, Hôpital la Timone, Département de Génétique Médicale, 13385 Marseille, France; nicolas.levy@univ-amu.fr; 6 Aix Marseille Université, APHM, CNRS, INSERM, CRCM, Multidisciplinary Oncology & Therapeutic Innovations Department, 13385 Marseille, France; fabrice.barlesi@ap-hm.fr

**Keywords:** lamins, lung cancer, lung adenocarcinoma, microRNAs, miR-9

## Abstract

Lung cancer represents the primary cause of cancer death in the world. Malignant cells identification and characterization are crucial for the diagnosis and management of patients with primary or metastatic cancers. In this context, the identification of new biomarkers is essential to improve the differential diagnosis between cancer subtypes, to select the most appropriate therapy, and to establish prognostic correlations. Nuclear abnormalities are hallmarks of carcinoma cells and are used as cytological diagnostic criteria of malignancy. Lamins (divided into A- and B-types) are localized in the nuclear matrix comprising nuclear *lamina*, where they act as scaffolding protein, involved in many nuclear functions, with regulatory effects on the cell cycle and differentiation, senescence and apoptosis. Previous studies have suggested that lamins are involved in tumor development and progression with opposite results concerning their prognostic role. This review provides an overview of lamins expression in lung cancer and the relevance of these findings for disease diagnosis and prognosis. Furthermore, we discuss the link between A-type lamins expression in lung carcinoma cells and nuclear deformability, epithelial to mesenchymal transition, and metastatic potential, and which mechanisms could regulate A-type lamins expression in lung cancer, such as the microRNA miR-9.

## 1. Introduction

Lung cancer is one of the most frequent cancers and the leading cause of cancer-related mortality in developed countries [1,2]. Among non-small cell lung cancer (NSCLC), adenocarcinoma represents the most frequent histological type. The European Society for Medical Oncology (ESMO) recommends molecular profiling of lung adenocarcinoma cells to select targeted therapy. This characterization comprises epidermal growth factor receptor (*EGFR*), V-Ki-ras2 Kirsten rat sarcoma viral oncogene homologue (*KRAS*), v-Raf murine sarcoma viral oncogene homologue B1 (*BRAF*), human epidermal growth factor receptor 2 (*HER2*) mutation profiles, together with anaplastic lymphoma kinase (*ALK*) and C-ros oncogene 1 (*ROS1*) rearrangement, and *c-MET* amplification [3,4]. A genetic alteration is found in approximately 50% of the patients with lung adenocarcinoma [5]. Despite advances in treatment during the last decade (such as chemotherapy, tyrosine kinase inhibitors or immunotherapy), the prognosis of advanced stages of NSCLC still remains poor. In this context, understanding which factors are involved in the metastatic process is a major issue, in order to identify new biomarkers and to develop new therapeutic strategies. 

Morphological changes in the size and shape of the nucleus, which are frequently observed in carcinoma cells [6,7], are commonly used as cytological diagnostic criteria of malignancy [8]. These nuclear abnormalities are probably the cause or the consequence of proteins modifications entering in the constitution of the nuclear matrix and/or the nuclear envelope (NE). The NE includes an inner and an outer nuclear membrane (INM, ONM), and is interrupted by nuclear pores implicated in nucleocytoplasmic exchanges. A family of type V intermediate filaments proteins called ‘lamins’ is one of the main components of the nuclear matrix, including the nuclear *lamina*, which is a network of lamin filaments located underneath the INM. Lamins confer capacity of resistance in cellular deformability [8,9,10]. They also regulate chromatin organization, DNA replication, DNA repair, transcription, differentiation [11,12,13], and apoptosis [14]. Lamins are known to interact with cytoskeleton through INM SUN proteins inside the LINC (LInker of Nucleoskeleton and Cytoskeleton) complex and are crucial for mechanotransduction and mechanical stability [9,15]. Lamins are divided into A-type and B-type. Two major isoforms of A-type lamins (lamin A and lamin C, referred as lamin A/C) and two minor isoforms (lamin A delta 10 and lamin C2) are alternative splice variants of *LMNA* [16,17], while lamin B1 is encoded by *LMNB1*, and lamins B2 and B3 result from an alternative splicing of *LMNB2* [18].

Lamins proteins are composed of a central helicoid domain surrounded by tow globular parts in N- or C-terminal. The C terminal tail bears an NLS region as well as an immunoglobulin-like domain [19,20]. Whereas lamin C is directly produced as a mature protein, lamins A, B1, and B2 are generated as precursors called ‘prelamins’ that undergo 3 (lamin A) or 4 (B-type lamins) steps of maturation. These processes occur though the CaaX motif in C-terminal that is specific for each precursor. Lamins A, B1, and B2 share common first steps of maturation. As a start, a farnesyl group (15-carbon hydrophobic group) is added to the cysteine residue of the CaaX box. This phenomenon leads to the anchorage of these prelamins to the endoplasmic reticulum membrane or to the outer nuclear envelope. The ‘aaX’ amino acids are then cleaved by ZMPSTE24/FACE1 or Rce1/FACE2 proteases. As a third step, the cysteine residue goes through methylation performed by an isoprenylcysteine carboxymethyl transferase (ICMT). At that point, B-type lamins are mature, whereas prelamin A needs to experience a last maturation step. Indeed, ZMPSTE24/FACE1 removes the last 15 amino acids of the precursor leading to the release of a mature non farnesylated protein. Therefore, while B-type lamins remain attached to the nuclear envelope thanks to their farnesyl anchor where they participate to the composition of nuclear *lamina*, lamin A and lamin C are localized simultaneously to the *lamina* and the rest of nuclear matrix [8,21,22,23].

Among the lamins subtypes, B-type lamins have a ubiquitous expression and are considered essential for cell survival. The expression of A-type lamins, however, appears to be related to the state of cellular differentiation. They are generally expressed in well-differentiated cells, while undifferentiated cells or embryonic cells do not show detectable levels of A-type lamins [24,25,26,27,28]. Moreover, the proportion of A-type and B-type lamins in cells may vary depending on tissues, in relation with their elasticity [29,30]. 

Interestingly, mutations of lamins or partners’ genes cause a heterogeneous landscape of disease clustered under the name ‘laminopathies’ (http://www.umd.be/LMNA/) in which some of them are characterized by premature aging features [31]. As example, the Hutchinson–Gilford’s Progeria Syndrome (HGPS) is a premature aging syndrome mainly caused by the p.G608G mutation in *LMNA* exon 11 firstly described in 2003 [32]. This mutation leads to a deletion of 50 amino acids on prelamin A, including the cleavage site of FACE1/ZMPSTE24 protease, resulting in the abnormal persistence of a C-terminal farnesylated cysteine at the end of the maturation processing [32,33]. This abnormal protein, called ‘progerin’, remains thus anchored in the INM, generating nuclear abnormalities and severe nuclear dysfunctions leading to a premature senescence. Patients die prematurely (mean age 14.6 years) usually from cardiovascular complications. Attractively, whereas other premature aging diseases present cancer predisposition based on the failure of their DNA repair systems (bloom syndrome/xeroderma pigmentusom), HGPS patients do not exhibit such susceptibilities [34]. Furthermore, Fernandez and collaborators recently identified a tumor-protective function of BRD4 by studying HGPS model [35]. Thus, accumulation of abnormal persistent farnylated truncated prelamin A combine with other factors could prevent oncogenic development in these patients.

Additionally, previous studies have hypothesized that lamins are involved in the development and progression of tumors. Few studies highlighted B-type lamins variation at a protein level, without a link to prognosis. In contrast, several studies suggested that A-type lamins are involved in the development and progression of cancers (Table 1) [36]. Variations of localization or level of expression of lamin A and/or C were reported in several histological types. As described in Table 1, a decrease of A-type lamins expression was found in breast cancers, prostate, colon, ovary, gastric, or endometrial, associated with a poor prognosis, leading to decreased overall survival, increase of metastatic sites number, tumor aggressiveness, or disease recurrence [37,38,39,40,41,42]. Conversely, some studies identified a link between the increase of A-type lamins expression and the progression of the colorectal, prostate, and ovarian cancer [43,44,45]. In these cancers, an enhanced expression of A-type lamins was associated with higher stage tumors or a decreased overall survival (Table 1). These results showed that the role of lamins A and C is probably dependent of the context and cancer type and requires other studies to identify their role in the progression of cancer, according to the histological type, the mutational profile and the stage of the underlying disease. 

In this review, we discuss lamins expression variations in lung cancer, the impact of these findings for disease diagnosis and prognosis and which mechanisms could regulate A-type lamins expression in lung cancer, such as the microRNA miR-9.

## 2. Lamins’ Expression in Normal and Cancer Lung Tissues

Only few studies were dedicated to lamins expression in the normal respiratory epithelium (bronchial and/or alveolar cells) and in lung cancer. In normal lung tissue, lamins A/C are physiologically expressed in a subset of cells, according to their differentiation stage. In lung cancer, a wide range of A-type lamins’ expression or localization has been described, without a clear relation with the prognosis.

### 2.1. Physiological Lamins’ Expression

In 1997 and 2014, Broers et al. [14,27] have studied lamins’ expression from normal human lung tissues. The expression of A- and B-types lamins was investigated by immunostaining on lung tissue using a panel of specific antibodies for each lamin subtypes. The A-type lamins’ expression was most prominent in well-differentiated epithelial cells as these proteins were expressed in bronchial columnar cells, containing highly bronchial differentiated cells, and in pneumocytes, contrary to bronchial basal cells. These results are coherent with literature, A-type lamins being poorly expressed in non-differentiated cells.

Moreover, the authors observed that lamin B2 was expressed by all bronchial cells and alveolar pneumocytes, whereas lamin B1 expression was restricted to the bronchial basal cells and was not detected in bronchial columnar cells. Pneumocytes showed heterogeneous staining.

### 2.2. Lamins’ Expression Depending on Lung Cancer Histological Subtypes

In lung cancer, only few studies were devoted to a description of lamins A, C, and B1 expression in cell lines and in human tissues. In 1990s, Kaufmann and Broers’ teams showed that A-type lamins were expressed in non-small cell lung cancer (NSCLC) cell lines, but were absent or very weak in small cell lung cancer (SCLC) cell lines, with no variation of lamin B1 expression. According to Kaufmann’s study, lamin A and C expression was reduced of more than 80% in SCLC cell lines compared to NSCLC cell lines, using Western and Northern Blotting [46]. Moreover, after v-*ras* oncogene transfection in NCI-H249 SCLC cell line, which changed the phenotype of cells from SCLC to NSCLC, a 10-fold increase in lamin A and C levels was observed, associated with higher amounts of lamins A and C mRNAs. Similar levels of lamin B were observed regardless v-*ras* transfection [46]. In 1993, Broers et al. [47] confirmed these findings in twenty-two human lung cancer cell lines using immunocytochemical, immunoblotting, and Northern blotting analyses. Lamins A and C were not or partly expressed in 14 out of 16 SCLC cell lines, whereas all NSCLC cell lines displayed lamins A and C expression. They also showed that B-type lamins were expressed in all SCLC and NSCLC cell lines. Moreover, analysis of 46 frozen human lung cancer biopsies showed consistent results: none or very weak lamins A and C expression was observed in 87% of SCLC cases (13/15), unlike adenocarcinoma and squamous cell carcinoma, which all presented lamins A and C expression. Interestingly, an aberrant cytoplasmic localization of A-type lamins was described rather than expected nuclear staining in several samples of adenocarcinomas. This abnormal cytoplasmic localization of A-type lamins has also been described in some colon, gastric, and pancreatic cancers [14,48]. Concerning B-type lamins’ expression, all tissues from SCLC and NSCLC were positive even though some cases of lung adenocarcinoma showed a loss of their expression [47].

To go further in lamins A and C expression in lung adenocarcinoma, Machiels et al. [49] have studied their expression in three lung adenocarcinoma cell lines, using a monoclonal antibody directed against both lamins A and C, and one antibody which specifically recognized lamin A but not lamin C in immunofluorescence and immunoblotting experiments. In one cell line (GLC-A1), lamin A aggregates were observed throughout the nucleoplasm, while the nuclear *lamina* had a weak signal. Moreover, in this cell line, amounts of lamins A and C proteins, as well as mRNAs, were lower than in other cell lines and the ratio between lamin A and lamin C (A/C) mRNAs was 1/8, instead of 1/1 in other cell lines. Again, no evidence of B-type lamins expression variation was notified.

In 2017, our team analysed lamin A, C, and B1 expression in metastatic cells of lung adenocarcinoma from pleural effusions. Interestingly, a strong decrease of lamin A, but not of lamin C expression was observed in a group of patients [50]. The lamin ratio [ratio = Lamin A/(Lamin A + Lamin C)] established by Western Blot led to classify the patients according to lamin A expression. This ratio was about 10-fold reduced for about a third of patients who were thus considered as presenting a low lamin A expression. Concerning the other group of patients, lamin A expression was considered as preserved. No variation of lamin B1 or lamin C expression was observed between the two groups. Using flow cytometry, this decrease of lamin A expression was correlated with the lack of EMA (Epithelial Membran Antigen)/MUC1, an epithelial malignancy marker which is involved in the epithelial to mesenchymal transition (EMT) [51,52]. Moreover, contrary to Broers’ study [14], no aberrant cytoplasmic localization of A-type lamins was found. Interestingly, the expression of lamin A was inversely correlated with the number of metastatic sites; patients with low lamin A expression had a higher number of metastases, and association of pleural, bone, and lung metastatic localization was significantly more frequent. Finally, these patients also had a higher *Performans Status* score compared with patients with high lamin A expression. Based on these findings, we had proposed low lamin A (with conserved lamin C) expression in pleural metastatic cells from lung adenocarcinoma as a pejorative factor associated with the development of metastasis. To our knowledge, this work is the first to investigate lamins A, C, and B1 expression as potential biomarkers in lung adenocarcinoma cells from metastatic pleural effusion. Nevertheless, the molecular mechanisms linking a reduced lamin A expression to an enhanced metastatic potential in lung adenocarcinoma are still lacking. 

All together, these data suggest a preferential role of A-type lamins linked to lung cancer other the B-type lamins. Moreover, lamin A protein seems mainly affected in contrast with lamin C.

### 2.3. Potential Link between the Loss of A-Type Lamins and Nuclear Deformability and Metastatic Potential Enhancement

The factors driving differences in lamins’ expression and the associated consequences with the metastatic process are still poorly understood. Within *lamina*, lamins indirectly interact with other proteins of the cytoskeleton, in particular with actin microfilaments. Through these interactions and connections, the stiffness properties of *lamina* would be transmitted via the cytoskeleton up to the plasma membrane, generating a real network of mechanic-transduction, and conferring a final link between the cell nucleus and the extracellular matrix [53]. Because the nucleus is responsible for the main properties of mechanical resistance, a decrease of its rigidity would be at the origin of a loss of the mechanisms of resistance of the cell in its entirety [54]. Several publications have emphasized the role of A-type lamins in nucleus deformability and stiffness, showing that a decrease of A-type lamins expression was associated with nuclear changes, such as an increase of the nucleus deformability and a decrease of its capacities of resistance [10,15,55,56]. Pajerowski et al. showed a higher nuclear deformability of embryonic stem cells, rather than more differentiated cells, depending on A-type lamins’ expression [57]. More recently, Davidson et al. developed a new microfluidic device showing that A-type lamins-deficient fibroblasts exhibited increased nuclear deformability and more plastic nuclear deformations compared to wild-type fibroblasts [55]. In more detail, Lammerding et al. demonstrated that mouse embryonic fibroblasts lacking both lamins A and C, only lamin A, or only lamin B1 had severely reduced, mildly reduced, and normal nuclear stiffness, respectively, in comparison to wild type cells [10]. These consequences of lacking lamins A and C can also be illustrated at a physiological level by polynuclear neutrophils, which are highly specialized and differentiated cells having distensible multilobed nuclei which do not express A-type lamins. Their nuclear deformability allows them to migrate through narrow capillaries or small tissue opening [56].

During cancer cell invasion, tumour cells produce growth factors, angiogenic factors, matrix metalloproteases, and have to acquire mobility and deformability to migrate through spaces in extracellular matrix smaller than the size of the nucleus. To spread to other organs, cells must deform to migrate through very small spaces and embolize into the circulation. The cellular deformability highly depends on the nucleus, which is the largest and stiffest cellular organelle. As described above, the deformability of the nucleus is largely determined by A-type lamins’ expression, and their loss confers superior deformability to cancer cells, which could facilitate migration through solid tissues and, at last, would promote the metastatic process [58,59].

Concerning lung cancer, consistent results were obtained. Pajerowski et al. demonstrated that the loss of A-type lamins in lung adenocarcinoma cell line A549 enhanced their nuclear deformability compared to A549 cells expressing these lamins [57]. Moreover, A-type lamins knockdown in A549 cells induced similar compliance and deformability than adult hematopoietic stem cells. Another study confirmed the link between the nuclear deformability and a low expression of lamins A and C in lung cancer cells: indeed, Lu et al. [60] exposed A549 cells to the anti-tumour green tea extract, which was previously described to induce cell adhesion and decrease motility in these cells. Green tea extract exposition has been described to lead to a 2-fold increase of A-type lamins’ expression at protein as well as mRNA levels, with a dose-related effect and thus, resulting in cell motility decrease.

All these studies demonstrate that A-type lamins are key factors involved in nuclear deformability, and the loss of lamin A only or both lamins A and C expression probably enhances metastatic potential in lung cancer. To our knowledge, only one study has made the link between the isolated reduction of lamin A expression and the enhancement of metastatic potential in patients suffering from metastatic lung adenocarcinoma [50]. However, lamins A and C are both encoded by the *LMNA* gene, and produced by alternative splicing resulting in two main mRNAs, the prelamin A transcript and the lamin C transcript. The isolated decrease of lamin A expression could be the consequence of a regulation via splicing factors or by microRNAs (miRNAs) at the post-transcriptional level.

## 3. Potential Mechanisms of A-Type Lamins Regulation in Lung Cancer Implicating miR-9 

### 3.1. MicroRNAs

MicroRNAs (also called miRNAs or miRs) are small non-coding RNAs, containing 18 to 25 nucleotides, described for the first time in 1993 in *C. elegans* [61]. Genes encoding miRNAs could be localized in intergenic regions in the dependence of specific promotors. Alternatively, miRNA encoding sequence could be localized in introns or exons of other genes, with a transcription being dependent of the promotor of these genes. First, a primary transcript composed of more than 1kb, called ‘pri-miRNA’, is produced by transcription using RNA polymerase II. The pri-miRNA is then cleaved by the RNase III enzyme Drosha into a smaller precursor called ‘pre-miRNA’ [62], containing 70 nucleotides organized in a stem-loop structure. After its exportation into the cytoplasm through the nuclear-cytoplasmic pores, the pre-miRNA undergoes a last cleavage step by the endonuclease Dicer, resulting in production of an 18–25 nucleotide-long duplex miRNA [63]. One strand of this duplex is eliminated in the RISC complex (RNA-induced silencing complex). The second strand, considered as the mature miRNA, is then able to target messengers RNAs (mRNAs). The seed region, which is defined by the nucleotides 2 to 7 in the 5′ region of miRNAs, settles most frequently on the 3′UTR (untranslated transcript region) of the target mRNA by complementarity. This interaction leads to target mRNA degradation or more frequently to the inhibition of its translation depending on a perfect or partial hybridization, respectively [64]. Interestingly, one miRNA can bind to hundreds of target mRNAs and inversely, different miRNAs, sharing the same or almost identical seed sequence can target the same mRNA [65,66]. Moreover, mature miRNAs can also be re-imported into the nucleus, where they recognize promoters of target genes, and thus regulate their transcription [67]. miRNAs are since recognized as leading actors of the regulation of genic expression. 

### 3.2. MicroRNAs and Lung Cancer

Several miRNAs contribute to the development and the progression of several cancers by controlling, for example, cell growth, tissue differentiation, and apoptosis. These miRNAs can function as tumour suppressors or oncogenes, and belong to the so called ‘oncomiRs’ [68]. They are also known to repress major cancer-related genes and might be considered as useful tools for diagnosis and prognosis. Finally, some of them are even considered as new therapeutic targets leading to the development of a preclinical study (NCT01829971) [69,70]. 

In lung cancer, many studies are descriptive, by analysing miRNA expression profile in lung tumour, in comparison to normal adjacent tissue. For some of them, a link between deregulated miRNAs and pathways was established using in silico approaches [71,72,73]. Moreover, the main miRNAs involved in lung cancer have newly been summarized by Uddin et al. [74]. Two recent publications [71,72] identified a miRNA expression profile, in which the five members of the miR-200 family (miR-141, miR-200a, miR-200b, miR-200c, miR-429) were up- or down-regulated in lung cancer and particularly in lung adenocarcinoma. The miR-200 family plays a major role by silencing epithelial to mesenchymal transition (EMT). EMT is characterized by the upregulation of mesenchymal markers and is associated with the down-regulation of epithelial differentiation markers, such as E-cadherin [75,76]. EMT is correlated with invasive tumor metastases and poor prognosis because of an increase of cell motility and invasiveness [51,52,76,77]. Thus, miR-200a/b/c are considered as tumor suppressors by repressing the mRNA translation of ZEB1 and ZEB2 transcriptional factors, leading to E-cadherin expression silencing [78]. Conversely, Tian et al. [72], described an up-regulation of these miRNAs in lung adenocarcinoma tissues. Nevertheless, this study focused on stage I lung adenocarcinoma without analyzing the metastatic potential. Concerning miR-429, its up-regulation in lung adenocarcinoma cell lines was associated with cell proliferation and cell metastasis [79]. Moreover, circulating microRNAs from liquid biopsy have been proposed as diagnostic and/or prognostic tools in lung cancer [80]. Among them, elevated serum levels of miR-141, miR-200b, and miR-429 have been proposed as potential biomarkers for early diagnosis in this context [81,82], even though elevation of these microRNAs in blood samples might not be lung specific as it has also been reported in other cancers. 

### 3.3. miR-9 in Lung Cancer

Mature miR-9-5p (miR-9 as previous id) results from three miR-9 genes, named *miR-9-1, -2, and -3,* depending on their localization (chromosomes 1, 5, and 15, respectively). miR-9 has been shown to be involved in the carcinogenesis and the metastatic process of cancers, either as an oncogene or as a tumour suppressor depending on cancer type [83,84]. 

Sromec et al. [85] proposed the high plasmatic miR-9 expression as a biomarker of NSCLC. In this study, plasma samples were collected from healthy donors and from NSCLC patients before surgery, and 1 month and 1 year after. Interestingly, following tumour resection, miR-9 levels significantly decreased below the normal level. Thus, the following of miR-9 plasma level could reflect the presence of NSCLC and the systemic response after tumour resection. 

In NSCLC cell lines, several studies showed an up-regulation of miR-9 using miRNA microarray, RT-qPCR and bioinformatic analyses [86,87,88,89,90,91,92]. In primitive tumours of NSCLC, miR-9 expression was higher than that in adjacent normal tissue [93,94,95]. This up-regulation in primitive tumour was correlated to advanced tumour-node-metastasis (TNM 2009), tumour size, and lymph node metastasis [94]. Furthermore, a high level of miR-9 expression was significantly associated with poorer progression free survival and overall survival. 

Expression level of miR-9 has also been described to influence oncologic drug efficiency. Low miR-9 expression in SCLC cell lines led to an increased sensitivity to etoposide and topotecan [96]. In adenocarcinoma cell lines, ectopic overexpression of miR-9 decreased the growth inhibitory effect of erlotinib [93]. Moreover, in adenocarcinoma cell lines, erlotinib downregulated miR-9 expression in a dose-dependent manner. The suggested mechanism was an activation of DNA methylation due to Erlotinib, leading to *miR-9-1* transcription inhibition. DNA methylation is an epigenetic mechanism, occurring on CpG islands, responsible for transcriptional silencing. In resected NSCLC samples, methylation of miR-9 genes is frequently observed [97]. However, contradictory results were described concerning the prognostic role of miR-9 genes methylation associated to miR-9 inhibition in NSCLC [89,93,97,98]. Methylation of *miR-9-3* was associated with a shorter overall survival, in lung squamous cell carcinoma [89], whereas methylated miR-9 genes were proposed as favourable prognosis biomarkers in NSCLC [97].

Moreover, several studies showed that miR-9 directly mediated E-Cadherin down-regulation in breast [99,100], prostate [101], oesophageal [102], ovarian [103], hepatocellular [104], and lung [95] carcinoma. miR-9 targets *CDH1*, the E-Cadherin mRNA and downregulates E-Cadherin expression, leading to β-catenin signalling activation and cell motility and invasiveness increase [100]. E-Cadherin is a transmembrane calcium-dependent glycoprotein, which plays an important role in maintaining the polarity and the contact of epithelial cells. In cancer research, downregulation of E-cadherin has been shown to promote the metastatic process by allowing the dissociation of carcinomatous cells. A negative correlation between the loss of E-cadherin expression in cancer cells and the severity of the underlying disease has also been described [105,106,107]. These consequences of E-cadherin inactivation have been shown in different models, both in vivo and in vitro [108,109,110,111,112]. Loss of E-Cadherin expression in tumour tissues predicted poor overall survival in NSCLC patients [113,114] and was associated with lymph node metastasis [113]. Moreover, in E-cadherin-deficient mouse model with lung adenocarcinoma, accelerated cancer progression and decreased survival were observed, associated with more metastatic sites number and in vitro, elevated migration of adenocarcinoma cells derived from this model [115].

Interestingly, miR-9 is also known to down-regulate the expression of Metastasis-Associated Lung Adenocarcinoma Transcript 1 (MALAT-1) [116]. miR-9 inhibits MALAT-1 expression through its direct binding on two miRNA binding sites in the MALAT-1 sequence, leading to MALAT-1 degradation in the nucleus [116]. MALAT-1 is a 7 kb long non-coding nuclear RNA, which is over-expressed in lung adenocarcinoma and other NSCLC cells [117,118,119,120]. MALAT-1 has been associated with metastasis in NSCLC and is known as a poor prognostic marker for survival in lung adenocarcinoma [116]. Indeed, MALAT-1 targets genes associated with lung cancer metastasis, involved in cell migration, tumor growth [117,118] and it was suggested that MALAT-1 promotes lung cancer brain metastasis by epithelial to mesenchymal transition (EMT) induction [120]. In addition, all these studies concerning miR-9 and MALAT-1 expressions were performed on various cancer cell lines or primary tumors obtained from patients with stage I–III NSCLC, including adenocarcinoma, squamous cell carcinoma, or other large cell carcinoma. No study was performed on metastatic cells from patients with stage IV lung adenocarcinoma. Moreover, MALAT-1 can be a blood based biomarker for NSCLC as it is detectable and increased in peripheral human blood sample from patients compared to cancer-free controls [121].

### 3.4. miR-9 and A-Type Lamins

Two publications showed that miR-9 is physiologically and highly expressed in neural cells, in which it inhibits the expression of lamin A but not of lamin C, by directly targeting prelamin A mRNA [122,123]. In HGPS, this mechanism prevents progerin (abnormal farnesylated prelamin A) expression in neural tissue, which explains the normal cognitive development of patients affected by HGPS. Inversely, most organs and systems, in which miR-9 is slightly or not expressed, are affected by progerin accumulation. 

Moreover, as described above, miR-9 is known to regulate the expression MALAT-1 [116], which is also implicated in the regulation of several splicing factors, such as serine-arginine (SR)-rich splicing factors (SRSF) 1, 2 and 3 [124]. SRSF1, like SRSF6, controls *LMNA* pre-mRNA alternative splicing, leading to the production of lamin A and progerin in HGPS cells and during physiological aging in a less extend [21,125]. MALAT-1 interacts with SRSF1 and modulates its expression in in vitro models by controlling the ratio of phosphorylated to dephosphorylated pools of SRSF1 [124,126]. SRSF1 and SRSF6 have been proposed as proto-oncogenes as they are overexpressed in many cancers [127,128]. Particularly in lung adenocarcinoma, high expression of SRSF1 is associated with the presence of metastases, a more aggressive phenotype, and chemotherapy resistance [77]. In human lung adenocarcinoma cell lines, SRSF1 overexpression lead to EMT, with the loss of epithelial markers (e.g., E-Cadherin) along with the acquisition of mesenchymal markers (e.g., vimentin, fibronectin, and N-cadherin) [77].

**Figure 1:**
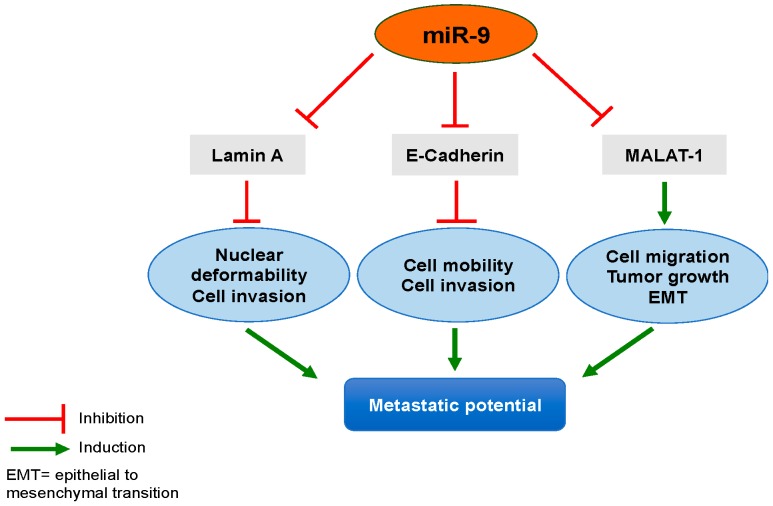
miR-9 as a potential central actor in the metastatic process of NSCLC. miR-9 was described to inhibit lamin A, E-cadherin, and MALAT-1 expression. Thus, miR-9 could indirectly regulate nuclear deformability, cell mobility, migration and invasion, tumor growth, and EMT, leading to the metastatic process in NSCLC.

In view of all these data, the oncomiR miR-9 seems to be a central actor in the carcinogenesis and the metastatic process of NSCLC. Indeed, through its direct regulation on E-cadherin, MALAT-1 and Lamin A, it could act at different levels to increase the metastatic potential (Figure 1). miR-9 is thus proposed as a biomarker associated to a poor prognosis in NSCLC patients, correlated to adverse clinical features and unfavourable survival. 

## 4. Conclusions and Future Perspectives

Previous works showed that A-type lamins’ levels were dramatically decreased in SCLC compared to NSCLC. In adenocarcinoma cells, A-type lamins’ aberrant cytoplasmic localization or reduced expression, with a more important decrease of lamin A expression compared to lamin C, were described. Furthermore, most published data result from old studies, only descriptive, which did not establish a link with the prognosis of the disease. A recent work of our team showed a reduction of lamin A expression, but not of lamin C, in lung adenocarcinoma metastatic cells obtained from pleural effusions. This isolated reduction of lamin A was associated with a higher number of metastatic sites and a poor performance status [50]. Interestingly, this expression profile of A-type lamins was also correlated with metastatic potential and a poor prognosis in other cancer types, such as ovarian cancer [38] and prostate carcinoma [40]. Thus, the loss of A-type lamins’ expression combined with the enhanced deformability of these cancer cells could confer them a high metastatic potential.

MiR-9 could be a good candidate to explain the reduction of lamin A expression in lung adenocarcinoma cells. We hypothesize that an up-regulation of miR-9 in cancer cells would inhibit lamin A expression without affecting lamin C expression, resulting to an increase of the nuclear deformability. Concomitantly, miR-9 would decrease the expression of the E-Cadherin, leading to EMT and allowing the dissociation of carcinomatous cells, increasing the metastatic potential. This hypothesis places miR-9 in the centre of the potential metastatic regulation by increasing nuclear deformability, cell invasion, and mobility and migration linked to EMT, through the direct targeting lamin A and E-Cadherin mRNAs, as well as the MALAT-1 lncRNA. Moreover, as EMT has also been associated with chemotherapy resistance [129,130,131], the reduction of lamin A expression, through miR-9 regulation, could represent a predictive factor of therapy efficacy.

Thus, future works on metastatic cells as well as on primitive tumour tissues from lung adenocarcinoma would facilitate investigation of the link between miR-9, lamin A, E-cadherin, and MALAT-1 expression. This would further our understanding of the mechanisms explaining metastatic potential and help with the proposal of new prognostic tools for patients with metastatic lung adenocarcinoma, and perhaps pave the way for the identification of new therapeutic targets in this devastating disease. 

## Figures and Tables

**Table 1:** Summary of A-type Lamins expression (at a protein level) depending on tumor type and link with the prognostic value.

Cancer/Tumor Type	A-Type Lamins Expression	Prognostic Value	References
***Gastric carcinoma***	Decrease	Decreased overall survival	Wu et al., *J. Exp. Clin. Cancer Res.* **2009**
	Cytoplasmic localization	/	Moss et al., *Gut* **1999**
***Breast carcinoma***	Decrease	Decreased overall survival	Capo-Chichi et al., *Chin. J. Cancer* **2011**
***Ovarian carcinoma***	Increase	Higher stage tumours	Wang et al., *J. Proteome Res.* **2009**
	Isolated decrease of lamin A	Decreased overall survival Increased number of metastatic sites	Gong et al., *Pathol. Res. Pract.* **2015**
***Endometrial carcinoma***	Isolated decrease of lamin A	Tumor agressiveness	Cicchillitti et al., *Oncotarget* **2017**
***Prostate adenocarcinoma***	Increase	/	Kong et al., *Carcinogenesis* **2012**
	Decrease	Increased risk for lymph node metastasis	Saarinen et al., *PLoS ONE* **2015**
***Colon carcinoma***	Increase	Decreased overall survival	Willis et al., *PLoS ONE* **2008**
	Decrease	Increase of disease recurrence	Belt et al., *EJC* **2011**
***Small Cell Lung carcinoma***	Decrease	/	Broers et al., *Adv. Exp. Med. Biol.* **2014** Broers et al., *Am. J. Pathol.* **1993** Kaufmann et al., *Cancer Res.* **1991**
***Lung adenocarcinoma***	Cytoplasmic localization	/	Broers et al., *Adv. Exp. Med. Biol.* **2014**
	Isolated decrease of lamin A	Increased number of metastatic sites Poor Performans status	Kaspi et al., *PLoS ONE* **2017**
